# Therapeutic value of tafamidis in patients with wild-type transthyretin amyloidosis (ATTRwt) with cardiomyopathy based on cardiovascular magnetic resonance (CMR) imaging

**DOI:** 10.1007/s00392-022-02035-w

**Published:** 2022-06-06

**Authors:** Bishwas Chamling, Michael Bietenbeck, Dennis Korthals, Stefanos Drakos, Volker Vehof, Philipp Stalling, Claudia Meier, Ali Yilmaz

**Affiliations:** 1 Department of Cardiology I, Division of Cardiovascular Imaging, University Hospital Münster, Albert Schweitzer Campus 1, A1, 48149 Münster, Germany; 2 Division of Electrophysiology, Department of Cardiovascular Medicine, University of Muenster, Albert-Schweitzer-Campus 1, Building A1, 48149 Münster, Deutschland

**Keywords:** Amyloidosis, Tafamidis, CMR, Mapping, ECV, Myocardial strain

## Abstract

**Objectives:**

The purpose of this study was to carefully analyse the therapeutic benefit of tafamidis in patients with wild-type transthyretin amyloidosis (ATTRwt) and cardiomyopathy (ATTRwt-CM) after one year of therapy based on serial multi-parametric cardiovascular magnetic resonance (CMR) imaging.

**Background:**

Non-sponsored data based on multi-parametric CMR regarding the effect of tafamidis on the cardiac phenotype of patients with ATTRwt-CM are not available so far.

**Methods:**

The present study comprised *N* = 40 patients with ATTRwt-CM who underwent two serial multi-parametric CMR studies within a follow-up period of 12 ± 3 months. Baseline (BL) clinical parameters, serum biomarkers and CMR findings were compared to follow-up (FU) values in patients treated “with” tafamidis 61 mg daily (*n* = 20, group A) and those “without” tafamidis therapy (*n* = 20, group B). CMR studies were performed on a 1.5-T system and comprised cine-imaging, pre- and post-contrast T1-mapping and additional calculation of extracellular volume fraction (ECV) values.

**Results:**

While left ventricular ejection fraction (LV-EF), left ventricular mass index (LVMi), left ventricular wall thickness (LVWT), native T1- and ECV values remained unchanged in the tafamidis group A, a slight reduction in LV-EF (*p* = 0.003) as well as a subtle increase in LVMi (*p* = 0.034), in LVWT (*p* = 0.001), in native T1- (*p* = 0.038) and ECV-values (*p* = 0.017) were observed in the untreated group B. Serum NT-proBNP levels showed an overall increase in both groups, however, with the untreated group B showing a relatively higher increase compared to the treated group A. Assessment of NYHA class did not result in significant intra-group differences when BL were compared with FU, but a trend to improvement in the treated group A compared to a worsening trend in the untreated group B (∆*p* = 0.005).

**Conclusion:**

As expected, tafamidis does not improve cardiac phenotype in patients with ATTRwt-CM after one year of therapy. However, tafamidis seems to slow down cardiac disease progression in patients with ATTRwt-CM compared to those without tafamidis therapy based on multi-parametric CMR data already after one year of therapy.

## Introduction

Amyloidosis is a family of multifaceted, heterogeneous diseases based on abnormally folded proteins and characterised by pathological accumulation of insoluble, polymeric protein fibrils in the extracellular space of various tissues and organs—sometimes leading to organ dysfunction, organ failure, and even death. The most common forms of amyloidosis infiltrating the human heart (cardiac amyloidosis, CA) are (a) immunoglobulin light chain (AL) [1] and (b) transthyretin amyloidosis (ATTR) [2] which in turn comprises two subtypes: a hereditary form (ATTRv) caused by the presence of a TTR gene mutation and a wild-type form (ATTRwt) caused by age-related instability of wild-type TTR [3]. Transthyretin, a physiological protein synthesised by the liver, misfolds into insoluble B-pleated sheets and accumulates as amyloid fibrils in the extracellular space of the myocardium resulting in a specific cardiomyopathy (CM) that is characterised by myocardial thickening and stiffening [4].

Until the recent approval of tafamidis, a TTR stabiliser, there were no specific treatment options for those with isolated ATTR-CM and the mortality rate of patients with the diagnosis of ATTR-CM was mentioned to be up to 64% after 5 years (Connors et al., Circulation 2016; 133(3):282–290). In the respective randomised and placebo-controlled ATTR-ACT study [5], it could be demonstrated that therapy with tafamidis for about 30 months reduced both all-cause mortality and cardiovascular-related hospitalizations as compared to placebo in patients with ATTR-CM. Other promising drugs for treatment of ATTR-CM such as the gene-silencers patisiran [6] and inotersen [7] were even shown to reduce cardiac amyloid load in some recent studies [8, 9], but so far are only approved for the treatment of neuropathy in ATTRv patients.

Unfortunately, tafamidis therapy is a highly expensive treatment [10], and identification and pre-selection of those patients with ATTR-CM who will benefit from such a treatment is challenging. Moreover, appropriate and valid assessment of therapy response during tafamidis treatment is also challenging, since an “improvement” in e.g. cardiac imaging markers or serum biomarkers was not shown in the respective ATTR-ACT study—and a meaningful “deceleration of progression” is difficult to assess due to the variability of this disease. In a recent study, Fontana et al. showed that cardiovascular magnetic resonance (CMR)-based non-invasive measurement of myocardial extracellular volume fraction (ECV) may be a welcome and suited imaging parameter for assessment of cardiac amyloid load that was shown to even decrease following treatment with patisiran in patients with ATTR-CM [9].

Multi-parametric CMR has been established as an important diagnostic tool for non-invasive detection as well as characterisation of cardiac amyloidosis: based on CMR techniques such as late-gadolinium-enhancement (LGE)-imaging [11], pre-/post-contrast T1-mapping with subsequent ECV calculation [12] and feature tracking (FT) for strain analysis [13], CMR allows to depict both the pattern and the extent of ATTR-CM [14, 15]. So far, there are neither prospective nor retrospective adequate studies that evaluated the effect of tafamidis on ATTR-CM based on multi-parametric CMR. In the present study, we aimed to evaluate the short-term effect of tafamidis on ATTR-CM using serial multi-parametric CMR studies in patients with ATTRwt.

## Methods and materials

### Patient characteristics and study design

In the present single-centre, retrospective study, we carefully looked at clinical, laboratory and CMR imaging data of 40 patients with ATTR amyloidosis (34 male/6 female, 77 ± 5 years) and presence of cardiac manifestation (ATTR-CM) who were/are treated and monitored using standardised procedures at our specialised “cardiac amyloidosis unit” at the University Hospital Muenster, Germany. All patients enrolled to the present study had either histologically proven ATTR amyloidosis and/or positive bone scintigraphy (in addition to positive CMR) in the absence of a monoclonal gammopathy, thereby proving the presence of cardiac ATTR. A hereditary form of ATTR (ATTRv) was ruled out by genetic analyses and only patients with wild-type ATTR (ATTRwt) were included. Patients with ATTR-CM were excluded if there was (a) a new diagnosis of cancer, (b) a new onset of terminal kidney disease with dialysis, (c) a new onset of atrial fibrillation, (d) a device implantation with continuous RV stimulation creating large image artefacts or (e) if they were older than 85 years.

All study patients underwent serial monitoring using multi-parametric CMR as a routine diagnostic tool with a follow-up time of 12 ± 3 months and were categorised into group A = treated with tafamidis 61 mg once daily (*N* = 20, 18 male/2 female, 76 ± 5 years) and into group B = without tafamidis treatment (*N* = 20, 16 male/4 female, 79 ± 5 years). Tafamidis treatment was started following the first CMR study entering this analysis in group A. Gene-silencers such as patisiran and inotersen were neither used prior to this study nor during follow-up of this study. Cardiac biomarker (NT-proBNP, N-terminal pro brain natriuretic peptides) as well as renal parameters (eGFR, estimated glomerular filtration rate) were obtained on the day of CMR examination. A tailored history taking focussing on subjective improvement in daily performance in comparison to the last visit was performed on regular basis. Based on a Chronic Heart Failure Questionnaire (CHQ), New York Heart Association (NYHA) classification was routinely assessed.

During the analysed study period, tafamidis 61 mg daily was initially started in 25 patients with ATTR-CM. However, five patients receiving tafamidis were excluded from this analysis due to either new diagnosis of cancer (*N* = 1), onset of terminal kidney disease with dialysis (*N* = 2), new onset of atrial fibrillation (*N* = 1) or device implantation with continuous RV stimulation creating large image artefacts (*N* = 1). The control group B consisting of 20 ATTR-CM patients without tafamidis treatment comprised those patients with ATTR-CM who did not receive tafamidis either due to individual unwillingness (*N* = 15) and/or medicolegal reasons (*N* = 5).

A new onset of atrial fibrillation in the time period between the two analysed CMR studies was an exclusion criterion for this study. Noteworthy, there were *N* = 5 patients with atrial fibrillation in group A (with tafamidis) and *N* = 4 patients in group B (without tafamidis). Since both CMR studies were performed in atrial fibrillation in these patients, we did not exclude them. Moreover, there were *N* = 3 patients per group in whom meaningful mapping analyses were not possible due to poor image quality—mostly due to atrial fibrillation. Nevertheless, functional and structural analyses based on e.g. cine- and LGE-imaging could also be performed in almost all patients with atrial fibrillation.

### CMR acquisition, T1 and ECV measurement

CMR studies were performed on a 1.5-T system (Ambition, Philips Healthcare, Best, The Netherlands). CMR data acquisition was performed according to the standardised protocols suggested by the Society for Cardiovascular Magnetic Resonance (SCMR) [16]. Our CMR protocol comprised a cine steady-state free precession pulse sequence for ventricular function and a two-dimensional (2D) inversion recovery fast spoiled gradient-echo sequence 10 to 15 min after administration of a gadolinium-based contrast agent (Gadobutrol 0.15 mmol/kg) for detection of myocardial pathology as described earlier [17]. Image analysis and interpretation was performed using commercially available software (cvi42—version 5.12.0, Circle Cardiovascular Imaging, Calgary, Alberta, Canada). Analysis of ventricular volumes and function as well as LV mass was made by contouring short-axis cine images.

In addition, a modified Look-Locker inversion recovery (MOLLI) T1-mapping sequence was applied in basal, mid and apical short-axes prior to contrast agent administration and ~ 20 min thereafter to determine native T1 and extracellular volume fraction (ECV) values as described previously [17]. T1-mapping and ECV were assessed and reported based on the consensus statement of SCMR. Motion corrected native and post-contrast T1-maps were generated from the pre- and post-contrast MOLLI-sequences. Motion corrected and segmented ECV maps were generated from the native and post-contrast segmented T1-maps, using the patient’s haematocrit level as described by us elsewhere [17]. “Global” T1 and ECV values were calculated by averaging all 16 segments from three short-axis slices.

### Feature tracking analysis

For the assessment of global LV deformation, three-dimensional (3D) LV global longitudinal strain (LV-GLS) derived from feature tracking (FT) was obtained using a validated algorithm integrated in the analysis software [18]. Landmarks for LV base (at the mitral valve ring) and apex were defined at end-diastole in all long-axis slices. Endocardial and epicardial borders were manually contoured in the end-diastolic frame in the three long-axis slices and in three short-axis slices, the most basal slice without through-plane distortion from the LV outflow tract, a mid-ventricular and an apical slice. Both the landmarks and the contours were automatically propagated throughout the cardiac cycle and manually corrected in case of inaccuracies. Subsequently, relative apical longitudinal strain (LS) was calculated based on the following equation: average apical LS/(average basal LS + mid LS), as defined by Phelan et al. [19].

### Statistical analysis

Due to the relatively small sample size, all data were non-normally distributed and therefore, non-parametric tests were used. Most variables are presented as medians and interquartile ranges (median ± interquartile range), few other parameters like ejection fraction (EF) and strain are expressed as change (%) from baseline (BL)—also mentioned in Tables 1, 2. Differences between groups were calculated with the Mann–Whitney *U* test, while the Wilcoxon signed-rank test was used for parameter changes over time in one group. For the assessment of appropriate relations between CMR parameters and clinical data and/or probable confounders Spearman's rank correlation was used. Statistical analysis was performed with SPSS (version 27.0, IBM Corp., Armonk, NY). A *p* value < 0.05 was considered statistically significant. All Figures were drawn by Prism Version 8 (GraphPad Software, La Jolla, USA).

**Table 1:** Baseline patient characteristics

Parameter	Group A = with tafamidis	*n*	Group B = without tafamidis	*n*	*p* value
Age (years)	76 (73–81)	20	80 (75–82)	20	0.18
Males/females	18/2	20	15/5	20	0.43
BMI (kg/m^2^)	25 (24–28)	20	25 (23–29)	20	0.90
eGFR (CKD-EPI) (mL/min/1.73 m^2^)	58 (47–67)	20	60 (54–68)	20	0.61
NYHA class	3 (2.0–3.0)	20	2.0 (1.3–2.0)	20	**0.006**
NT-proBNP (pg/ml)	2068 (1646–3267)	16	1810 (1260–2858)	16	0.19
NAC (National Amyloidosis Centre) Staging Score					
Stage I	12	20	16	20	0.31
Stage II	5		2		
Stage III	3		2		
Medication					
*ß*-blockers	No. of patients	(No.)	No. of patients	(No.)	
Metoprolol	4	3	8	4	0.69
Bisoprolol	9		6		
Carvedilol	1		0		
Nebivolol	0		1		
Diuretics	No. of patients	(No.)	No. of patients	(No.)	
Loop diuretics	13	7	11	6	0.74
Thiazides and thiazide-like	3		1		
Potassium-sparing	6		5		
Major CMR findings					
LV-EF (%)	51 (48–58)	19	57 (50–61)	20	0.14
LV-EDVi (ml/m^2^)	91 (76–102)	19	81 (77–98)	20	0.90
LV mass index (g/m^2^)	104 (90–120)	20	90 (82–104)	20	**0.046**
Max. LV thickness (mm)	20 (18–22)	20	20 (17–21)	20	0.29
RV-EF (%)	49 (44–52)	20	54 (46–61)	20	0.17
RV-EDVi (ml/m^2^)	91 (75–101)	20	80 (73–102)	20	0.74
3D global longitudinal peak strain (%)	– 7.30 (– 7.70 to – 5.00)	18	– 8.60 (– 9.33 to – 7.45)	20	**0.012**
Apical/(basal + mid) strain ratio (3D), *n*	0.81 (0.79–1.11)	18	0.83 (0.67–0.90)	20	0.19
Global native T1 [950–1050 ms]	1111 (1094–1125)	17	1097 (1077–1126)	17	0.33
Basal septal native T1 [950–1050 ms]	1110 (1101–1127)	17	1096 (1049–1128)	17	0.43
Global ECV [25–31%]	57 (51–62)	17	56 (48–60)	17	0.51
Septal ECV [25–31%]	64 (52–70)	17	57 (48–66)	17	0.15

All data are given as median (interquartile range), if not mentioned otherwise. Units are mentioned in small brackets (). Normal range of values are mentioned in large brackets []

*BMI *body mass index, *eGFR (CKD-EPI) *estimated glomerular filtration rate according – chronic kidney disease epidemiology collaboration, *NYHA *New York Heart Association, *NT-proBNP *N-terminal pro brain natriuretic peptides, *CMR *cardiovascular magnetic resonance, *LV *left ventricle, *RV *right ventricle, *EF *ejection fraction, *EDVi *enddiastolic volume index, *ECV *extracellular volume fraction

*p* < 0.05 is considered as significant

**Table 2:** Change in biomarkers and CMR findings over a follow-up period of 1 year

Parameter	Group A = with tafamidis (*N* = 20)			Group B = without tafamidis (*N* = 20)			∆*p* value
	Baseline	Follow-up	*p* value	Baseline	Follow-up	*p* value	
NYHA	3 (2.0–3.0)	2.0 (2.0–3.0)	0.10	2.0 (1.3–2.0)	3 (1.3–3.0)	**0.007**	**0.005**
NT-proBNP (pg/ml)	2068 (1646–3267)	2403 (1857–4112)	**0.046**	1810 (1260–2858)	2614 (1059–3526)	**0.011**	0.20
Major CMR findings							
LV-EF (%)	51 (48–58)	51 (49–57)	0.75	57 (50–61)	51 (47–56)	**0.003**	**0.041**
LV-EDVi (ml/m^2^)	91 (76–102)	88 (78–103)	0.72	81 (77–98)	85 (76–92)	0.17	0.16
LV mass index (g/m^2^)	104 (90–120)	106 (89–114)	0.32	90 (82–104)	92 (86–112)	**0.034**	**0.028**
Max. LV thickness (mm)	20 (18–22)	20 (17–22)	0.77	20 (17–21)	20 (18–22)	**0.001**	**0.005**
RV-EF (%)	49 (44–52)	48 (41–52)	0.06	54 (46–61)	49 (43–54)	**0.010**	0.72
RV-EDVi (ml/m^2^)	91 (75–101)	102 (79–109)	**0.006**	80 (73–102)	85 (74–100)	**0.96**	**0.014**
3D global longitudinal peak strain (%)	– 7.30 (– 7.70 to – 5.00)	– 6.70 (– 7.10 to – 5.60)	0.58	– 8.60 (– 9.33 to – 7.45)	– 7.30 (– 9.08 to – 5.08)	**0.009**	0.46
Apical/(basal + mid) strain ratio (3D), *n*	0.83 (0.67–0.90)	0.88 (0.69–0.96)	0.18	0.81 (0.79–1.11)	0.89 (0.8–1.07)	**0.001**	**0.005**
Global native T1 [950–1050 ms]	1111 (1094–1125)	1111 (1088–1131)	0.25	1097 (1077–1131)	1116 (1095–1126)	**0.038**	**0.025**
Basal septal native T1 [950–1050 ms]	1110 (1101–1127)	1104 (1093–1121)	0.29	1096 (1049–1128)	1095 (1078–1123)	0.30	0.15
Global ECV [25–31%]	57 (51–62)	54 (48–63)	0.19	56 (48–60)	57 (49–62)	**0.017**	**0.016**
Septal ECV [25–31%]	64 (52–70)	63 (54–68)	0.57	57 (48–66)	60 (51–69)	**0.007**	**0.021**

All data are given as median (interquartile range), if not mentioned otherwise. Units are mentioned in small brackets (). Normal range of values are mentioned in large brackets []

*CMR *cardiovascular magnetic resonance, *LV *left ventricle, *RV *right ventricle, *EF* ejection fraction, *EDVi *enddiastolic volume index, *ECV *extracellular volume fraction, **∆***p* significance between the changes among treated and untreated patients in respective parameters

*p* < 0.05 is considered as significant

## Results

### Baseline (BL) patient characteristics

Details of BL clinical parameters as well as multi-parametric CMR findings are shown in Table 1. Parameters like age (*p* = 0.18), sex (*p* = 0.43), body mass index (BMI) (*p* = 0.90), eGFR (*p* = 0.60), left ventricular ejection fraction (LVEF) (*p* = 0.14) and NT-proBNP (*p* = 0.19) showed no significant difference between the two groups. Only clinical assessment of NYHA classification resulted in slightly higher NYHA class at BL in the tafamidis group A (*p* = 0.006). Different CMR parameters—apart from 3D-LV-GLS–also showed similar results between both groups where 3D-LV-GLS [– 7.30 (– 7.70 to – 5.00) vs – 8.60 (– 9.33 to – 7.45); *p* = 0.012]—showed a minor, however, significant difference at BL indicating a slightly advanced disease stage in the tafamidis group A (in accordance with the aforementioned NYHA results). Tafamidis was well tolerated by all patients in group A. There were no serious adverse events during the assessment period as a result of which therapy had to be discontinued.

### Longitudinal assessment of CMR-based volumetric parameters

**Fig. 1:**
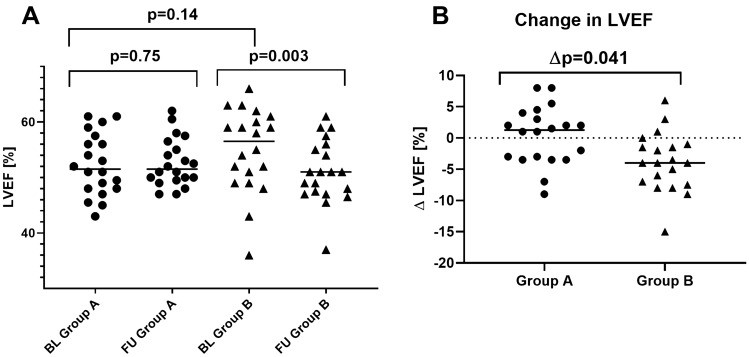
Graph illustrating the differences in left ventricular ejection fraction (LV-EF) between BL vs FU in group A and B (**A**) as well as comparison of the extent of change in LV-EF between group A and B (**B**); *p *value < 0.05 is considered as significant. *BL *baseline, *FU *follow-up

**Fig. 2:**
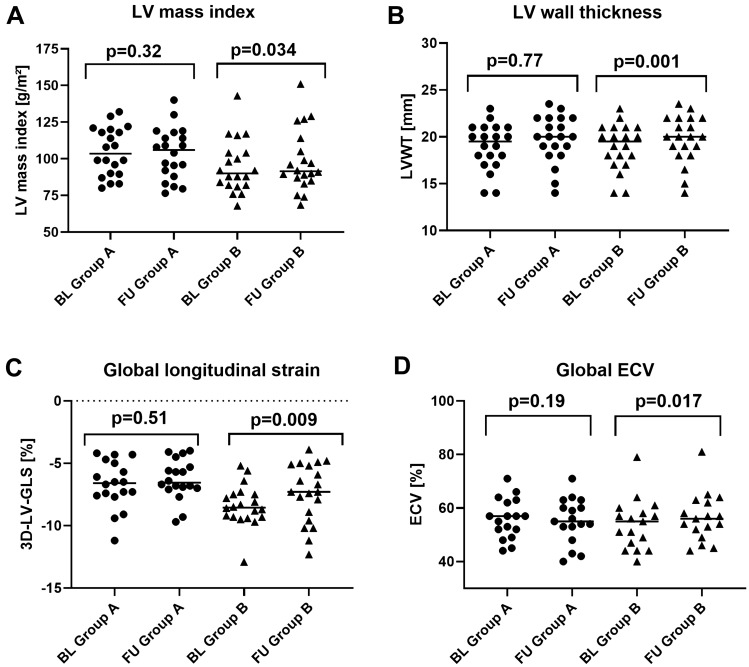
Graph illustrating changes from BL to FU in both groups regarding **A** left ventricular mass index (LVMi), **B** left ventricular wall thickness (LVWT), **C** three-dimensional (3D) LV global longitudinal strain (LV-GLS) derived from feature tracking, **D** extracellular volume fraction (ECV); *p *value < 0.05 is considered as significant. Horizontal line represents group medians. *BL *baseline, *FU *follow-up

**Fig. 3:**
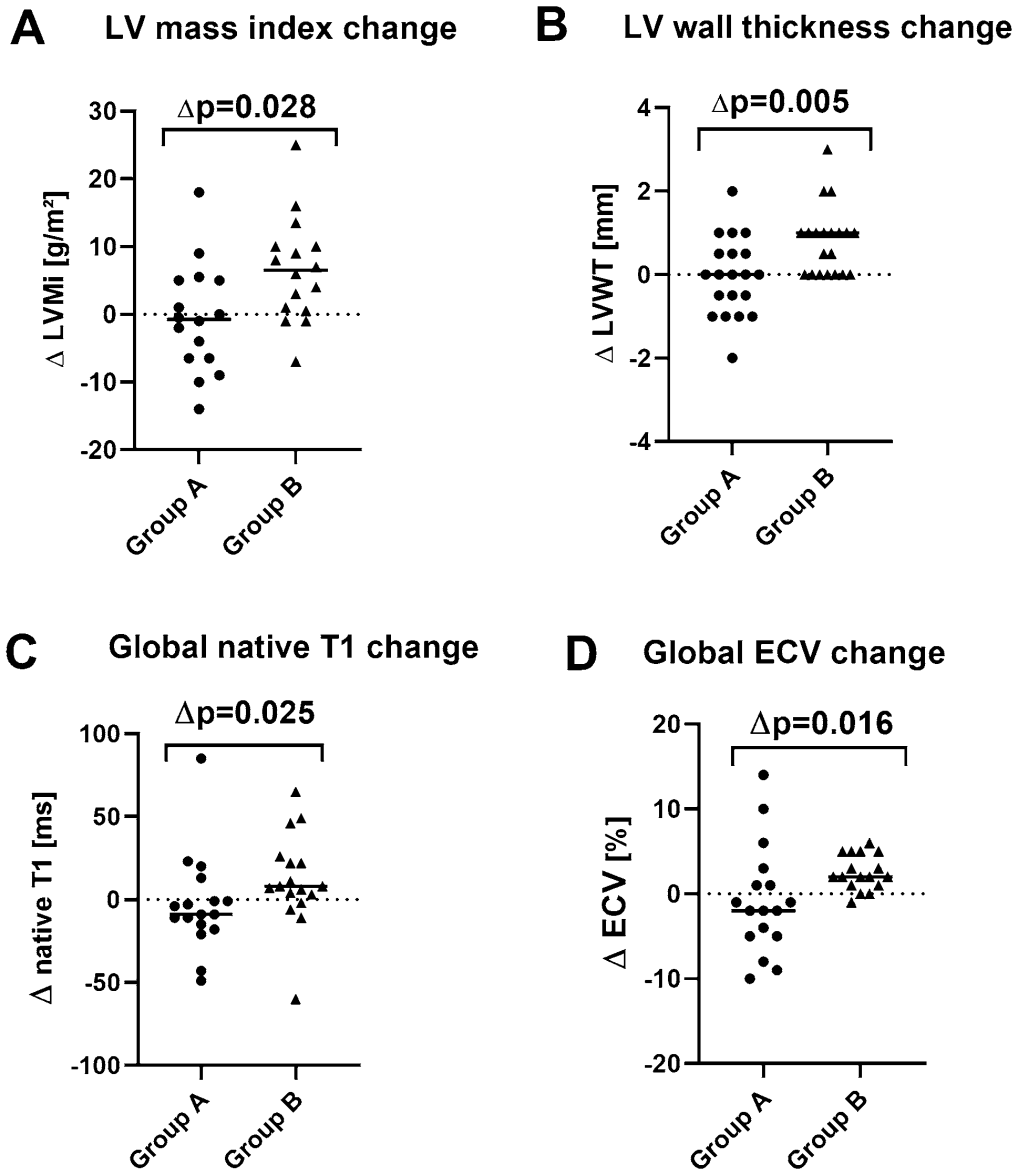
Graph illustrating longitudinal changes from BL to FU in group A and group B patients with their respective *p *values with respect to **A** left ventricular mass index (LVMi), **B** left ventricular wall thickness (LVWT), **C** global T1 values, **D** global extracellular volume fraction (ECV); *p *value < 0.05 is considered as significant. Horizontal line represents group medians

An overall deterioration of LVEF was observed in group B at 1 year-FU [57% (50–61%) vs 51% (47–56%), *p* = 0.003], whereas in the tafamidis group A neither significant improvement nor worsening of LVEF was observed when FU values were compared to BL [51% (48–58%) vs 51% (49–57%), *p* = 0.75; Fig. 1A]. Moreover, all CMR volumetric parameters—except right ventricular ejection fraction (RVEF) and right ventricular enddiastolic volume index (RVEDVi)—remained rather stable at FU in the tafamidis group A (Table 2). Only RVEF and RVEDVi showed a slight decrease during tafamidis treatment. In contrast, major volumetric CMR parameters in group B showed a minor, however, concurrent worsening during FU [Fig. 2A–B]. Accordingly, significant differences between delta values of group A and group B were observed for LVEF (∆*p* = 0.041, Fig. 1B), LVMi (∆*p* = 0.03, Fig. 3A), LVWT (∆*p* = 0.005, Fig. 3B). The remaining CMR volumetric parameters like LVEDVi (*p* = 0.157) and RVEF (*p* = 0.718) showed no differences.

### Longitudinal assessment of CMR-based T1-mapping and ECV parameters

As illustrated in Table 2, BL values of native T1 in the myocardium were increased in both groups. Noteworthy, there was no significant difference regarding BL native T1 values between both groups [1111 ms (1094–1125 ms) in group A vs 1097 ms (1077–1126 ms) in group B, *p* = 0.33]. Longitudinal intra-group assessment showed no substantial difference between BL and FU native T1 values in both groups. However, inter-group comparison of change in native T1 values between BL and FU resulted in a significantly higher increase in group B compared to group A (∆*p* = 0.025, Fig. 3C).

Accordingly, global myocardial ECV was elevated in both groups already at BL [57% (51–62%) in group A vs 56% (48–60%) in group B; normal range: 25–31%], but no significant difference was observed between both groups at BL (*p* = 0.51). Longitudinal intra-group assessment showed no significant change in myocardial ECV in group A [BL vs FU: 57% (51–62%) vs 54% (48–63%), *p* = 0.19]. In contrast, there was a significant increase in both global as well as septal ECV values in group B [global ECV: 56% (48–60%) vs FU: 57% (49–62%); *p* = 0.017; Fig. 2D], [septal ECV: 57% (48–66%) vs 60% (51–69%); *p* = 0.007]. Similar to our T1-mapping findings, inter-group comparison regarding the longitudinal change in global ECV showed a significant increase in group B compared to group A (∆*p* = 0.016) as shown in Fig. 3D.

### Longitudinal assessment of CMR-based strain parameters

3D-LV-GLS was reduced in both groups at BL [– 7.30% (– 7.70 to – 5.00%) in group A vs – 8.60% (– 9.33 to – 7.45%) in group B] with a minor, however, significant difference between both groups. The most pronounced impairment of left ventricular regional longitudinal strain (LV-RLS) was measured in the basal segments in both groups [− 4.9% (– 6.0 to – 2.95%) in group A vs – 6.5% (– 8.0 to – 5.45%) in group B]. The “apical-to-(basal + midventricular)”-ratio of LVRLS (reflecting the degree of apical sparing) was 0.81 (0.79–1.11) in group A vs 0.83 (0.67–0.90) in group B (*p* = 0.012) as illustrated in Table 1.

Longitudinal intra-group assessment showed no significant change in 3D-LV-GLS [– 7.30% (– 7.70 to – 5.00%) at BL vs – 6.70% (– 7.10 to – 5.60%) at FU; *p* = 0.58] or apical sparing [0.83 (0.67–0.90) at BL vs 0.88 (0.69–0.96) at FU; *p* = 0.16] in the tafamidis group A. In contrast, there was a significant worsening in 3D-LV-GLS [– 8.60% (– 9.33 to – 7.45%) vs – 7.30% (– 9.08 to – 5.08%); *p* = 0.009; Fig. 2C] and in apical sparing [0.81 (0.79–1.11) vs 0.89 (0.8–1.07); *p* = 0.001] in group B. Assessment of longitudinal inter-group changes showed no relevant differences in 3D-LV-GLS between both groups (∆*p* = 0.46), however, a significant worsening in apical sparing in group B compared to group A (∆*p* = 0.005) as shown in Table 2.

### Clinical assessment of disease progression (based on NT-proBNP and NYHA status)

In spite of a concurrent increase in NT-proBNP serum values in both groups [BL vs FU: 2068 (1646–3267) pg/ml vs 2403 (1857–4112) pg/ml in group A and 1810 (1260–2858) pg/ml vs 2614 (1059–3526) pg/ml in group B], group B without tafamidis showed a higher increase in NT-proBNP compared to group A, however, without statistical significance [∆NT-proBNP: + 486 pg/ml vs + 869 pg/ml, ∆*p* = 0.20].

Clinical symptoms were assessed based on NYHA classification and showed a trend towards improvement in group A without reaching statistical significance compared to a trend towards significant worsening in groups B, (*p* = 0.096 in group A vs *p* = 0.007 in group B). Importantly, assessment of longitudinal inter-group changes regarding change in clinical NYHA status demonstrated a significant difference between both groups (∆*p* = 0.005) with the aforementioned trend to improvement only in the tafamidis group A. Detailed analysis of each patient showed that six patients in group A showed an improvement in NYHA class, two patients a worsening of symptoms whereas the remaining twelve patients stayed unchanged. In contrast, clinical deterioration based on NYHA class was observed in eight patients in group B whereas the remaining patients of this group showed stable NYHA status. Details of NYHA classification and NT-proBNP measurements can be found in Table 2.

## Discussion

**Fig. 4:**
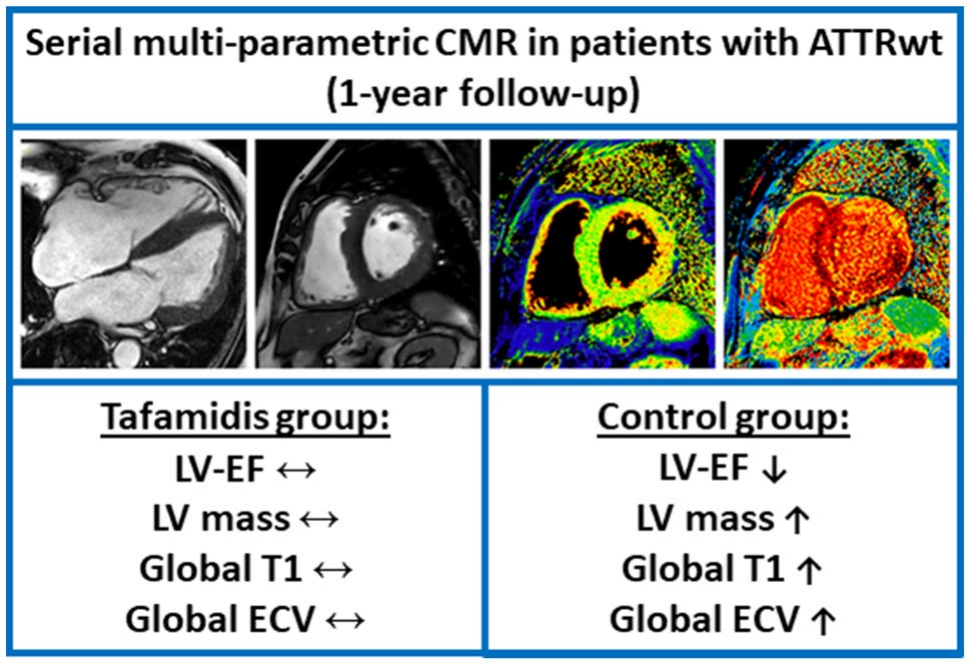
CMR-based changes in ATTR-CM after 1 year tafamidis therapy. Example of cardiovascular magnetic resonance (CMR) images obtained in a patient with ATTRwt-CM showing cine-images in long- and short-axis views as well as corresponding native T1- and extracellular volume fraction (ECV) maps in a basal short-axis view. Whereas cardiac status war rather preserved after 1 year of tafamidis therapy, the control group without tafamidis showed a worsening of different CMR-based parameters

Considering (a) some limitations of the tafamidis approval study ATTR-ACT, (b) current challenges in the selection of those patients with ATTR-CM who will benefit from a tafamidis therapy, (c) current difficulties regarding appropriate assessment of tafamidis therapy response and (d) the high costs of tafamidis therapy in patients with ATTR-CM, our present (investigator-initiated and non-sponsored) results should be important from a clinical point-of-view (Fig. 4): First, tafamidis therapy in patients with ATTR-CM did not essentially improve cardiac disease status—that was carefully assessed by multi-parametric CMR—after one year of therapy, but slowed down cardiac disease progression compared to those patients with ATTR-CM who did not receive tafamidis. Second, a trend towards improvement in clinical symptoms based on NYHA class was only observed in patients with ATTR-CM receiving tafamidis therapy, however, this beneficial NYHA trend was not accompanied by a concurrent decrease in serum NT-proBNP levels.

When we take a first superficial look on the results of the tafamidis approval study ATTR-ACT [5], we will see that therapy with tafamidis for about 30 months reduced even hard clinical endpoints (all-cause mortality and cardiovascular-related hospitalizations) as compared to placebo in patients with ATTR-CM. However, when we take a closer look, we will recognise that despite a hazard ratio (HR) of 0.69 regarding the reduction in all-cause mortality by tafamidis therapy in that ATTR-ACT study, the respective 95%-confidence interval regarding this HR was quite broad (0.49–0.98) and quite close to 1 (!). Hence, there was not a “substantial” added value of tafamidis with regard to all-cause mortality—but only a “little” added value (at the expense of high tafamidis costs). Consequently, the approval of tafamidis 61 mg for the treatment of ATTR-CM e.g., in Germany was primarily due to “clinical improvements” (e.g. regarding walking distance in 6 min walk test, 6-MWT) following tafamidis therapy. But once again, if we take a closer look on the 6-MWT results of the ATTR-ACT study, we will see that tafamidis treatment did not improve 6-MWT results, but rather slowed down the natural decrease in walking distance measured by a 6-MWT—but did not increase the respective walking distance. Hence, it is quite difficult to assess and determine the clinical benefit of tafamidis therapy based on anyway worsening 6-MWT results in individual patients. Therefore, 6-MWTs are not performed on a regular level at our institution.

Taken together, we do need other trustable, valid and robust parameters that allow accurate monitoring of tafamidis (and other therapeutic) effects on the human heart in patients with ATTR-CM—with subsequent assessment of therapy response [20]. Since data obtained from transthoracic echocardiography during the aforementioned ATTR-ACT study did not help to identify those patients showing a therapeutic benefit [5], we decided to use multi-parametric CMR in patients with ATTR-CM to carefully assess both functional and structural cardiac changes during tafamidis treatment [14, 17]. And since tafamidis treatment is highly expensive in case of ATTR-CM and withholding tafamidis in patients with ATTR-CM is ethically not justifiable at least in those patients who fulfil the inclusion/exclusion criteria of the ATTR-ACT study, we were forced to pursue a “retrospective” study design as detailed in the methods section. However, since we have established standardised procedures regarding diagnosis and follow-up of ATTR-CM at our centre, we were able to collect standardised and robust “cardiac” data. Moreover, we were able to collect comparative data in patients with ATTR-CM who did not receive tafamidis either due to individual unwillingness and/or medicolegal reasons.

**Fig. 5:**
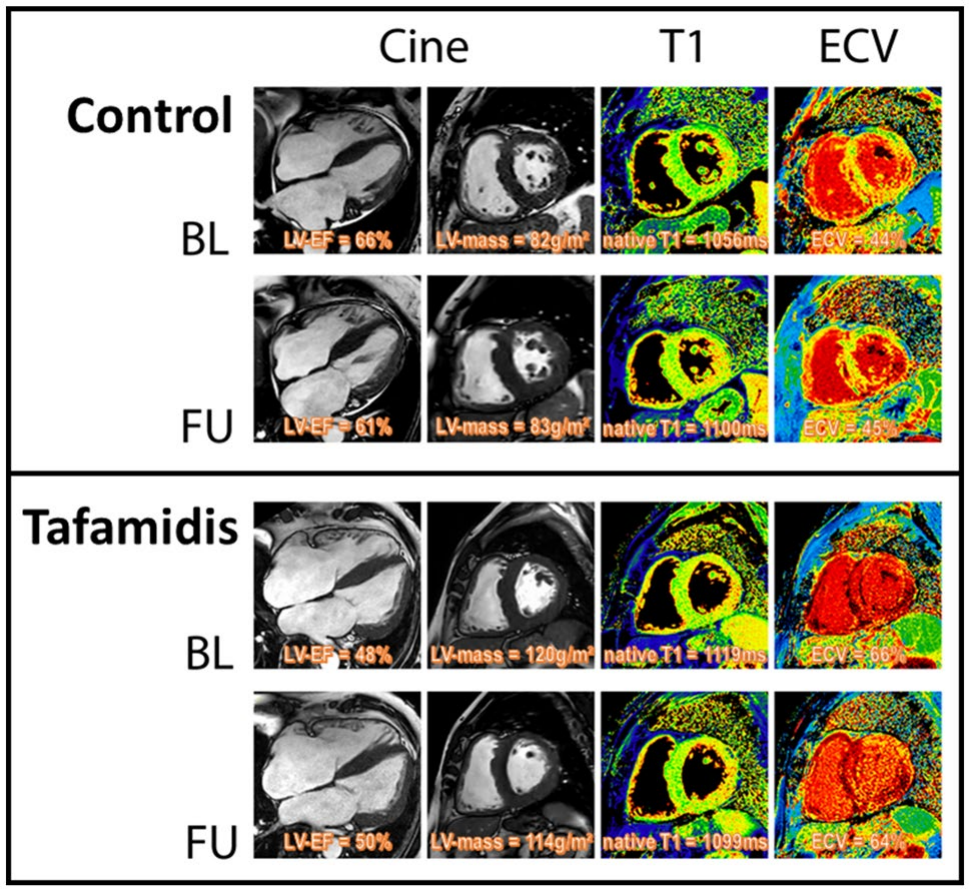
Example of cardiovascular magnetic resonance (CMR) images obtained at baseline (BL, upper panel) and follow-up (FU, second panel) in a patient with ATTRwt-CM from the control group B showing cine-images in long- and short-axis views in diastole as well as corresponding native T1- and extracellular volume fraction (ECV) maps in a basal short-axis view. In comparison, lower panels show CMR images obtained in a patient from the tafamidis group A at BL (third panel) and FU (bottom panel). A subtle reduction of highly elevated myocardial ECV values in the septal as well as lateral wall segments was observed in the group A patient receiving tafamidis whereas constant or slightly increased ECV values were measured in the group B patient without tafamidis

Our present CMR results clearly indicate that cardiac function deteriorates (e.g. decrease in LV-EF and RV-EF) and cardiac amyloid load increases (e.g. increase in LVMi as well as ECV) already after one year of monitoring in those patients with ATTR-CM who did not receive tafamidis and whose cardiac disease course rather reflects “natural” disease progression. Based on the respective results of this study, we may even deduce some valuable numbers regarding the “natural” cardiac disease course that can be used for comparison in future studies: e.g. a decrease in LV-EF of up to 8%, a decrease in RV-EF of up to 5%, an increase in LVMi of up to 7 g/m^2^ or an increase of septal ECV of up to 4%—each within one year of follow-up (Fig. 5). Noteworthy, these observations are (in principle) in line with the results of Fontana et al. that were observed in their respective “control” group that was compared to the treatment group with patisiran, recently [9]. In contrast to the patisiran effect that was observed by Fontana et al., treatment with tafamidis 61 mg did not improve cardiac disease status, but slowed down cardiac disease progression, and seems to prevent (amongst others) a decrease in LV-EF or RV-EF, a worsening in GLS, an increase in LV mass or an increase in T1-mapping based ECV (if measured by multi-parametric CMR) already after one year. In this context, recent suggestions made by experts of this field regarding imaging parameters that should be used to monitor cardiac disease progression or regression in case of ATTR-CM may need to be carefully consider the present data [21, 22].

Moreover, a trend towards improvement in clinical symptoms based on NYHA class was only observed in patients with ATTR-CM receiving tafamidis therapy in the present study. Noteworthy, this beneficial NYHA trend was not accompanied by a concurrent decrease in serum NT-proBNP levels. Hence, it needs to be emphasised once again that NT-proBNP reflects “current” cardiac volume status as well as intra-cardiac pressure load, and may either quickly increase or decrease (within a few days) if the respective volume/pressure status changes—due to e.g. new onset of atrial fibrillation or intensification of diuretic therapy. Therefore, the serum marker NT-proBNP is not an ideal biomarker to accurately assess the “underlying cardiac disease burden” in case of ATTR-CM. Obviously, this needs to be done by more accurate and comprehensive imaging modalities such as multi-parametric CMR. Moreover, well-known ATTR staging algorithms that are (amongst other parameters) based on NT-proBNP values [23] need to be used with caution and should be extended by CMR imaging parameters in the future. However, since our present study was not blinded, we cannot rule out a placebo effect that may have caused or at least contributed to the improvement in NYHA class in our group A (with tafamidis) compared to group B (no tafamidis) without a concurrent improvement in NT-proBNP.

## Study limitations

This study has several limitations. First, the sample size was relatively small and this was not a prospective, randomised, controlled trial. However, considering the fact that ATTR-CM is still a rare disease and that a prospective, non-sponsored, investigator-initiated study is impossible due to financial as well as ethical issues, our retrospective, however, standardised approach should be appropriate. Second, patients were treated only with tafamidis if they fulfilled the inclusion and exclusion criteria of the ATTR-ACT study at the time of first presentation. Third, standardised data from other disciplines and methods (e.g. neurological examination or bone scintigraphy) were not available and therefore not considered in the present analysis. Finally, future CMR studies with a larger study size and a longer follow-up time are needed to further improve identification of those patients that will benefit from tafamidis therapy, since our present limited data showed a non-neglectable individual variation of therapy response and do not allow detailed correlation and regression analyses to detect trustable CMR “predictors” for tafamidis therapy response.

## Conclusion

As expected, tafamidis does not improve cardiac phenotype in patients with ATTRwt-CM after one year of therapy. However, tafamidis seems to slow down cardiac disease progression in patients with ATTRwt-CM compared to those without tafamidis therapy based on multi-parametric CMR data already after one year of therapy.

## Abbreviations

ATTRwt: Wild-type transthyretin amyloidosis
CM: Cardiomyopathy
CMR: Cardiovascular magnetic resonance
BL: Baseline
FU: Follow-up
LV-EF: Left ventricular ejection fraction
ECV: Extracellular volume fraction
LVMi: Left ventricular mass index
LVWT: Left ventricular wall thickness
LGE: Late-gadolinium-enhancement
NT-proBNP: N-terminal pro brain natriuretic peptides
eGFR: Estimated glomerular filtration rate
NYHA: New York Heart Association
CHQ: Chronic Heart Failure Questionnaire
LV-GLS: Left ventricular global longitudinal strain
RVEF: Right ventricular ejection fraction
RVEDVi: Right ventricular enddiastolic volume index
